# Applicability of the CT Radiomics of Skeletal Muscle and Machine Learning for the Detection of Sarcopenia and Prognostic Assessment of Disease Progression in Patients with Gastric and Esophageal Tumors

**DOI:** 10.3390/diagnostics14020198

**Published:** 2024-01-16

**Authors:** Daniel Vogele, Teresa Mueller, Daniel Wolf, Stephanie Otto, Sabitha Manoj, Michael Goetz, Thomas J. Ettrich, Meinrad Beer

**Affiliations:** 1 Department of Diagnostic and Interventional Radiology, Ulm University Medical Center, 89081 Ulm, Germany; teresa.mueller@uni-ulm.de (T.M.); daniel.wolf@uni-ulm.de (D.W.); sabitha.manoj@uni-ulm.de (S.M.); michael.goetz@uniklinik-ulm.de (M.G.); meinrad.beer@uniklinik-ulm.de (M.B.); 2 Visual Computing Group, Institute for Media Informatics, Ulm University, 89081 Ulm, Germany; 3 XAIRAD—Artificial Intelligence in Experimental Radiology, University Hospital of Ulm, 89081 Ulm, Germany; 4 Comprehensive Cancer Center Ulm (CCCU), Ulm University Medical Center, 89081 Ulm, Germany; stephanie.otto@uniklinik-ulm.de; 5 Department of Internal Medicine I, Ulm University Medical Center, 89081 Ulm, Germany; thomas.ettrich@uniklinik-ulm.de; 6 i2SouI—Innovative Imaging in Surgical Oncology Ulm, University Hospital of Ulm, 89081 Ulm, Germany; 7 MoMan—Center for Translational Imaging, Department of Internal Medicine II, University Hospital of Ulm, 89081 Ulm, Germany

**Keywords:** sarcopenia, CT radiomics, machine learning, prediction, gastroesophageal cancer

## Abstract

Purpose: Sarcopenia is considered a negative prognostic factor in patients with malignant tumors. Among other diagnostic options, computed tomography (CT), which is repeatedly performed on tumor patients, can be of further benefit. The present study aims to establish a framework for classifying the impact of sarcopenia on the prognosis of patients diagnosed with esophageal or gastric cancer. Additionally, it explores the significance of CT radiomics in both diagnostic and prognostic methodologies. Materials and Methods: CT scans of 83 patients with esophageal or gastric cancer taken at the time of diagnosis and during a follow-up period of one year were evaluated retrospectively. A total of 330 CT scans were analyzed. Seventy three of these patients received operative tumor resection after neoadjuvant chemotherapy, and 74% of the patients were male. The mean age was 64 years (31–83 years). Three time points (t) were defined as a basis for the statistical analysis in order to structure the course of the disease: t1 = initial diagnosis, t2 = following (neoadjuvant) chemotherapy and t3 = end of the first year after surgery in the “surgery” group or end of the first year after chemotherapy. Sarcopenia was determined using the psoas muscle index (PMI). The additional analysis included the analysis of selected radiomic features of the psoas major, quadratus lumborum, and erector spinae muscles at the L3 level. Disease progression was monitored according to the response evaluation criteria in solid tumors (RECIST 1.1). CT scans and radiomics were used to assess the likelihood of tumor progression and their correlation to sarcopenia. For machine learning, the established algorithms decision tree (DT), K-nearest neighbor (KNN), and random forest (RF) were applied. To evaluate the performance of each model, a 10-fold cross-validation as well as a calculation of Accuracy and Area Under the Curve (AUC) was used. Results: During the observation period of the study, there was a significant decrease in PMI. This was most evident in patients with surgical therapy in the comparison between diagnosis and after both neoadjuvant therapy and surgery (each *p* < 0.001). Tumor progression (PD) was not observed significantly more often in the patients with sarcopenia compared to those without sarcopenia at any time point (*p* = 0.277 to *p* = 0.465). On average, PD occurred after 271.69 ± 104.20 days. The time from initial diagnosis to PD in patients “with sarcopenia” was not significantly shorter than in patients “without sarcopenia” at any of the time points (*p* = 0.521 to *p* = 0.817). The CT radiomics of skeletal muscle could predict both sarcopenia and tumor progression, with the best results for the psoas major muscle using the RF algorithm. For the detection of sarcopenia, the Accuracy was 0.90 ± 0.03 and AUC was 0.96 ± 0.02. For the prediction of PD, the Accuracy was 0.88 ± 0.04 and the AUC was 0.93 ± 0.04. Conclusions: In the present study, the CT radiomics of skeletal muscle together with machine learning correlated with the presence of sarcopenia, and this can additionally assist in predicting disease progression. These features can be classified as promising alternatives to conventional methods, with great potential for further research and future clinical application. However, when sarcopenia was diagnosed with PMI, no significant correlation between sarcopenia and PD could be observed.

## 1. Introduction

Sarcopenia is the progressive and generalized loss of muscle mass and strength with age. The manifestation of this primary aging process may be exacerbated by comorbidities, malnutrition and physical inactivity [1,2]. Sarcopenia may also be present in children and adolescents—for example, in the setting of tumor diseases such as hepatoblastoma, long-term steroid therapy, muscular dystrophy, or chronic liver disease [3,4,5]. In addition to functional limitations, this often means an increase in trauma/falls and resulting injuries for those affected, which in turn can further reduce quality of life. Sarcopenia affects about 20% of all women and men in the seventh decade of life and about half of all women and men in the 75-year-old age group [6]. Potential risk factors include malnutrition, lifestyle factors (obesity, physical inactivity, smoking, extreme sleep duration), inflammatory processes and tumor diseases.

Various radiological methods are available for the diagnosis of sarcopenia, such as dual X-ray absorptiometry (DXA) or the sectional imaging methods computed tomography (CT) and magnetic resonance imaging (MRI). CT, the most widely used cross-sectional imaging technique, can be used to analyze skeletal muscle on various body regions. In addition to muscle area or volume, density values of individual muscles or muscle groups can be determined [7]. Usually, the segmentation of the muscles and the determination of the cross-sectional area (CSA) are performed at the L3 or L4 level. The psoas muscle alone or the entire muscle area at the corresponding height is analyzed at this level [8]. The muscle areas obtained from a CT slice correlate very well with the total body muscle mass [9,10]. In most cases, the muscle area results are correlated with body size and the so-called skeletal muscle index (SMI) is determined (SMI = CSA/height^2^). The advantage of these indices is that in all CT examinations of the trunk, the muscle mass can be recorded in addition to the primary clinical question, and corresponding calculations can be performed (secondary use of CT data). CT is particularly suitable for diagnosing sarcopenia in tumor patients, as it is often performed at the time of diagnosis and subsequently at defined intervals to evaluate the response to therapy.

### 1.1. Gastric and Esophageal Tumors

Sarcopenia, as a skeletal muscle disease with diagnostic criteria that have not been uniformly defined to date, is considered a prognostic risk factor for various tumor diseases. In a meta-analysis, Shachar et al. evaluated a total of 38 studies with 7843 patients with the diagnosis of a solid tumor [11]. The most common tumors investigated in the studies were hepatocellular carcinoma (*n* = 11), pancreaticobiliary tumors (*n* = 6), gastroesophageal tumors (*n* = 4), urothelial carcinomas, renal cell carcinomas, and colorectal carcinomas (*n* = 3 each). In all included studies, a low SMI determined at diagnosis was shown to be a negative prognostic factor for survival, for patients with metastasis as well as for patients without metastasis. Patients with esophageal or gastric tumors take a central role due to the unfavorable combination of the prominent tumor location and a costly and debilitating therapy. It has been shown that these facts can lead to an unfavorable risk situation, increased post-operative complication rate and reduced overall survival [12,13].

### 1.2. Radiomics

In contrast to common practice, which focuses on the visual assessment and interpretation of radiological images including skeletal muscles, the term radiomics stands for the conversion of quantitative image features—so-called radiomic features—into data sets that are not tangible to the eye. Radiomic features are both shape features of a defined region of interest [14], such as volume or sphericity, and higher-order features that reflect the distribution of voxel values or the arrangement of different voxel intensities in the ROI [15]. Together with powerful computational technologies and artificial intelligence (AI)-based image analysis the field of radiomics was further developed [16,17]. With data characterization algorithms diagnostic image patterns can be detected and converted into quantitative data [18,19,20]. An AI-based image analysis addresses the challenges of evaluating a great number of features together with their impact on tumor progression.

Until now, radiomics has mainly been applied in oncologic patients to extract tumor features in order to visualize tumor heterogeneity and assess prognosis [21,22]. In addition, muscle segmentation and adipose tissue content were investigated to analyze muscle quality in tumor patients [23,24]. In their study, Kitajima et al. could stratify the risk of oncological outcomes with a combined assessment between the pre-operative psoas muscle index and intramuscular adipose tissue [25]. In comparison to classical muscle segmentation, radiomic features offer more information for qualitative evaluation, providing more potential for evaluating qualitative patterns. Given the quantitative and additionally qualitative differences in skeletal muscles of patients with tumor diagnosis and muscle loss, radiomics might be helpful to detect sarcopenia and predict tumor progression. Therefore, only a few studies used radiomic features to analyze skeletal muscle and assess sarcopenia in cancer patients [26,27,28]. In their study, de Jong et al. used the CT radiomics of skeletal muscle to predict the response to chemotherapy in patients with stage IV non-small lung cancer (NSLC) [28].

### 1.3. Purpose of the Study

Overall, the results of this study should contribute to the development of an efficient means for the diagnosis of sarcopenia. In addition, the effect of sarcopenia on the outcome of patients diagnosed with esophageal or gastric cancer at the end of the considered period will be evaluated. This should form a basis for the classification of the influence of sarcopenia on the prognosis of patients with esophageal or gastric cancer. In addition, the importance of technical advances such as CT radiomics with regard to diagnostic and prognostic processes are to be investigated. For the first time, the prediction of sarcopenia using CT radiomics and machine learning is evaluated in our study.

Based on these findings, we hypothesized that:

(1) Sarcopenia detected with routine CT scans influences disease progression in patients with gastroesophageal cancer.
(2) The CT radiomics of skeletal muscle and machine learning can detect sarcopenia and predict tumor progression.

## 2. Material and Methods

This study was approved by the ethics committee of our institution. One hundred patients with a biopsy-proven diagnosis of esophageal or gastric cancer between January 2015 and December 2019 who underwent contrast-enhanced CT scans at our institution were identified for this retrospective study. Patients were excluded from the study if only one CT dataset or no pre-therapeutic baseline CT was available. After applying these criteria, a total of 83 patients were included. To characterize the final study cohort, patient demographic, clinical and imaging data were carefully collected from electronic medical records, the picture archiving and communication system (PACS) and the radiology information system (RIS) of our institution.

All 83 patients included in the study underwent (neoadjuvant) chemotherapy after their initial diagnosis. Seventy-three of these patients subsequently underwent surgery. In addition, three time points (t) were defined as a basis for the statistical analysis in order to structure the course of the disease. For the evaluation, the appropriate CT scan was assigned to each time point. This structure is intended to ensure the comparability of the time points and the selected CT scans within the patient cohort as far as possible. The following time points were defined: t1 = initial diagnosis, t2 = following (neoadjuvant) chemotherapy and t3 = end of the first year after surgery in the “surgery” group or end of the first year after chemotherapy in the “non surgery” group.

**Figure 1:**
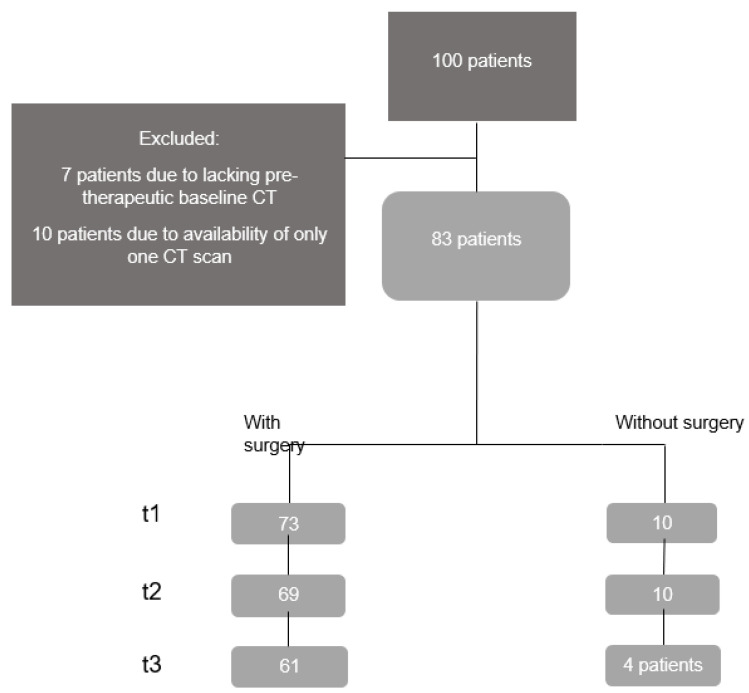
Patient recruitment for the study cohort.

Patient outcome, as the target parameter, was defined as the achieved stage at t3 according to the Response Evaluation Criteria In Solid Tumors (RECIST 1.1) [29]. Specifically, it was evaluated whether progressive disease (PD) was present despite tumor therapy or whether the therapy resulted in an improvement or stability (stable disease (SD) or partial remission (PR), or complete remission (CR)). Evaluation according to the response criteria was performed by a board-certified radiologist with more than 10 years of experience in oncologic imaging. The number of patients for whom CT scans were available changed from t1 to t2 to t3 during the period under consideration. At moment t1, the diagnosis was established. In the group that underwent surgery, the interval after neoadjuvant therapy or surgery was denoted as t2. The patients were followed up for either one year after surgery or one year after chemotherapy (t3). Figure 1 shows a flowchart of the study cohort selection. Due to the small number of patients in the “non surgery” group (*n* = 10), the focus of the described procedures was placed on the “surgery” group.

The data basis of this study was the archived CT data sets of the patient cohort, which originated from examinations during the initial diagnosis or subsequent routine staging examinations. Image acquisition was performed on a 256-channel scanner Philips Brilliance iCT (Philips, Eindhoven, The Netherlands) and a 128-channel Somatom Definition AS+ (Siemens Healthineers, Erlangen, Germany). The standard acquisition and reconstruction parameters included a tube voltage of 120 kV using an automatic modulated tube current (matrix of 512 × 512, in-plane resolution between 0.62 × 0.62 mm and 0.86 × 0.86 mm). CT scans were acquired after the intravenous contrast injection of Ultravist^®^ 370 (Bayer Schering Pharma, Berlin, Germany) at a weight-matched dose (1.1 mL/kg) followed by 60 mL of sodium chloride solution for the portovenous phase (with a delay of 90 s after starting injection) of the chest and abdomen. For the evaluation in the study, axial reconstructions were used with a section thickness of 3.0–5.0 mm.

### 2.1. Muscle Segmentation and Assessment of Sarcopenia

**Figure 2:**
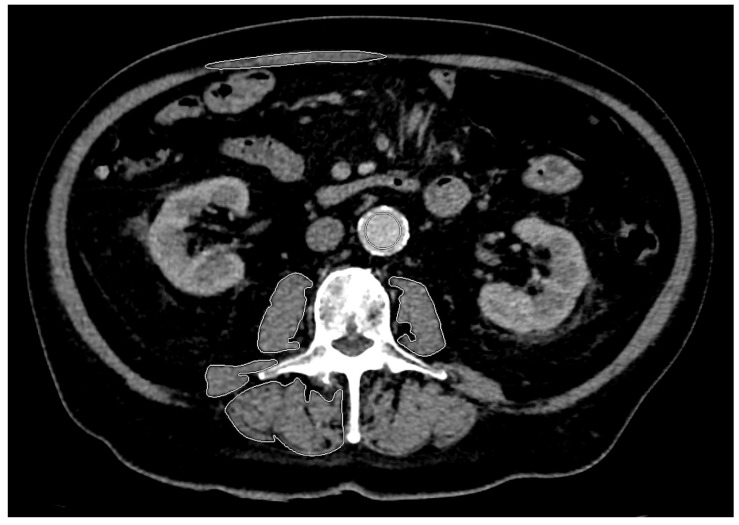
Axial CT at the L3 level and segmentation of the bilateral psoas major muscle, the right erector spinae muscle, quadratus lumborum muscle and rectus abdominis muscle. The latter was excluded from further analysis due to the high variance in segmentation. This semi-automatic segmentation of skeletal muscles was performed using mint Lesion™ software (version 3.8.4, mint Medical GmbH, Heidelberg, Germany). CT: computed tomography; L: lumbar.

In their study, Shen et al. described the highest correlation between the skeletal muscle area of a single slice image and the skeletal muscle mass of the whole body about 5 cm above the L4-L5 level [30]. Several studies proposed that the area of the psoas major muscle (TPA [cm^2^]) alone can also be used as a diagnostic and prognostic parameter [31,32,33]. To define and diagnose sarcopenia in the described patient cohort, the psoas muscle index (TPI [cm^2^/m^2^]) at the L3 level was collected following a validated method already used in other studies [34,35,36,37]. To maintain high reproducibility, the lowest sectional plane of L3, on which the right processus transversus was completely visible, was chosen following the definition of Perthen et al. [38]. First, the semi-automatic segmentation of the TPA was performed using mint Lesion™ software (version 3.8.4, mint Medical GmbH, Heidelberg, Germany). In addition to segmenting the psoas major muscle to define sarcopenia, the erector spinae muscle and quadratus lumborum muscle were also segmented for radiomic feature analysis. Figure 2 shows an example of skeletal muscle segmentation. To exclude distortions due to possible differences in the selected contrast-enhanced CT phase, the density value of the aorta at the selected section plane was collected as a reference value.

For the definition of sarcopenia, the threshold value of the lowest quartile of the TPI values was determined gender-specifically at t1, t2 and t3 in each case. This is consistent with the approach taken in numerous previous studies [34,35,37,39,40]. After assigning patients to the “sarcopenic” and “non-sarcopenic” groups, the percentage of patients considered sarcopenic in the patient collectives with and without surgery was recorded descriptively on a gender-specific basis at each of the three time points.

### 2.2. Statistics

The collected data were anonymized, exported, and stored in Microsoft Office Excel 365 program (version 17). The statistical analysis of these data was performed using IBM SPSS Statistics version 28.

The patient clinical and demographic data were evaluated descriptively to characterize the included patient cohort. To evaluate the effect of sarcopenia on patient outcomes after tumor therapy, the following procedures were performed: By means of an exact test according to Fisher, it was analyzed whether PD occurs more frequently in sarcopenic patients than in non-sarcopenic patients. Sarcopenic patients were considered separately at t1, t2, and t3. Using the odds ratio (OR), the quota ratio of the characteristics was calculated as an effect size measure. To test whether PD occurs more rapidly in sarcopenic patients than in non-sarcopenic patients, the number of days between the initial diagnosis and the onset of PD was calculated with the mean and SD for all affected individuals. Three separate Mann–Whitney U tests were used to test for statistically significant differences between the “sarcopenic” and “non-sarcopenic” patients.

### 2.3. Radiomics Model and Machine Learning

Skeletal muscle segmentation and radiomic feature extraction were conducted using mint Lesion™ (version 3.8.4, mint Medical GmbH, Heidelberg, Germany). Radiomic features were obtained through the discretization method of fixed bin number (FBN), utilizing a Bin Amount of 32. To accentuate regions of rapid intensity change, a Laplacian-of-Gaussian filter (LoG) was employed as a preprocessing step, employing a sigma value of 2. The aggregation method used for matrix computation was “3D average directions”. The radiomic features were extracted with a resample filter and voxel resampling size (X, Y, Z) set at 1 × 1 × 1 mm^3^. Additionally, a second-order distance of 1 was considered. The intensity calculation for radiomic features was restricted within a specific range using a threshold filter set at 0. An overview of the extraction parameters is shown in Table 1.

Radiomic features were assessed based on their distinct grayscale patterns within the segmented skeletal muscles, following the Image Biomarker Standardization Initiative (IBSI) guidelines [18]. A comprehensive set of eighty-five features was extracted from each analyzed skeletal muscle. These encompassed features concerning size, shape, first-order statistics depicting voxel intensity distribution within the specified region, and characteristics associated with the grayscale co-occurrence matrix. For an exhaustive list of the radiomic features utilized in model development, please refer to Table A1 in Appendix A.

**Figure A1:**
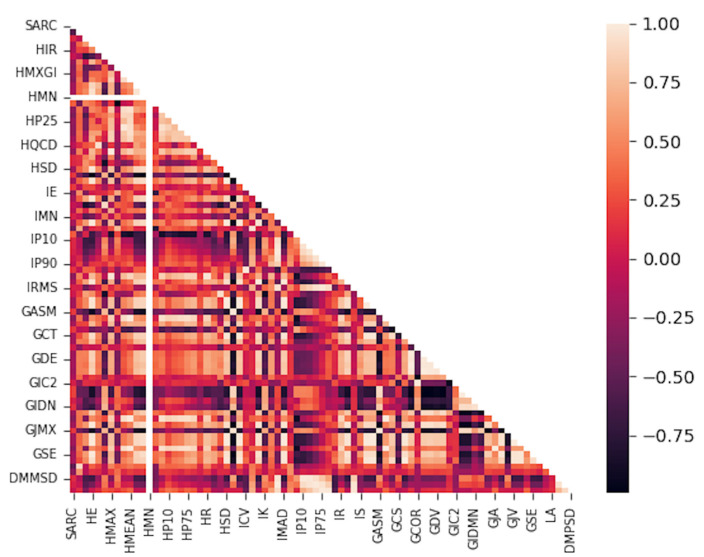
Correlation heatmap of radiomic features. SARC: sarcopenia; Radiomic features of first order: HIR: Histogram Interquartile Range, HMXGI: Histogram Maximum Histogram Gradient Intensity, HMN: Histogram Minimum, HP25: Histogram P25th, HQCD: Histogram Quartile Coefficient Dispersion, HSD: Histogram Standard Deviation, IE: Intensity Energy, IMN: Intensity Mean, IP10: Intensity P10th, IP90: Intensity P90th, IRMS: Intensity Root Mean Square; Radiomic features of second order gray-level co-occurrence matrix (GLCM): GASM: Angular Second Moment, GCT: Cluster Tendency, GDE: Difference Entropy, GIC2: Information Correlation 2, GIDN: Inverse Difference Moment Normalized, GJMX: Joint Maximum, GSE: Sum of Entropy; DMMSD: Density Mean Minus Standard Deviation.

To achieve greater generalizability, increased modeling potency, faster computation, and mitigating overfitting, we adopted an approach to select optimal features. As a first step, a Pearson correlation analysis was performed and we plotted the correlation heatmaps (Figure A1 in Appendix A). For highly correlated features (with a correlation coefficient ≥0.98), only one of the features was kept and the other one was dropped. This was followed by feature selection utilizing the logistic regression model employing the least absolute shrinkage and selection operator [41,42]. As the λ-value progressively increased, certain regression coefficients consistently decreased, approaching 0. Those variables retaining non-zero values emerged as the most effective predictors. The selected features are displayed in Table 2. The selected features were then provided to the machine learning models.

In order to test the validity of the radiomic features as predictive radiological parameters with regard to sarcopenia and tumor development, a workflow was carried out, which is also referred to in the literature as the “radiomics pipeline” and at the end of which models were developed using machine learning [43]. In the present study, models were developed to make predictions about the target parameters of sarcopenia and PD, as well as their correlation, based on the generated radiomic features of the segmented muscles. Specifically, the aim was to analyze whether CT radiomics could detect sarcopenia and predict tumor progression alone (PD) or in combination with sarcopenia (sarcopenia + PD). The goal was to evaluate the performance of the models using the metrics “Accuracy” and “Area under the curve” (AUC) in order to make statements about the diagnostic and prognostic value of the radiomic features [44]. To further process the data in the context of machine learning, the software library “scikit-learn” (version 1.1.3) in the Python programming language (version 3.10) was used [45,46]. The developed models are based on several commonly used algorithms, which are briefly listed below: Decision tree (DT) is a supervised learning algorithm, which is a tree-structured classifier. The internal nodes of the classifier represent the features of the dataset, and branches represent the decision rule and each leaf denotes the outcome. [47]. The K-Nearest Neighbor (KNN) procedure classifies new data based on their similarity to existing data [48]. During the training phase, the algorithm stores the entire training dataset as a reference. When making predictions KNN computes the distance between the input data sample and all the training samples. Then algorithm identifies the K nearest neighbors and assigns the most common class label among the K neighbors as the predicted label. In a “Random Forest” (RF), the outputs of several decision trees are combined and merged into one result via a so-called “Majority Voting” [47]. The RF algorithm makes use of a collection of decision trees to make the predictions. The algorithm creates multiple decision trees using different random subsets of data samples and features. The prediction from each decision tree is computed, and based on the majority votes of prediction, it predicts the final output. Since this sometimes resulted in asymmetric group sizes, subgroups with fewer examples were artificially enlarged, if necessary, by means of “resampling” using the “Adaptive Synthetic Sampling Approach for Imbalanced Learning”, according to their degree of difficulty in learning, in order to preferably generate a balanced data set as the basis for the machine learning models [49].

To evaluate the performance of the models, 10-fold cross-validation was used [50]. For this purpose, the data sets of the defined groups were mixed and randomly divided into ten groups of equal size. In ten runs, one of the groups was alternately determined as the test group according to a ratio of 90%/10%, and the respective model was trained with the remaining nine groups. The trained model was then presented with the test data as previously unknown data in order to evaluate its performance, i.e., its ability to generalize, and to exclude so-called “overfitting”. The mean and standard deviation of the “accuracy” and the “area under the curve” (AUC) over the ten runs are presented, each evaluated on the test data sets. In addition to the tabular presentation of the values obtained, some results were presented graphically as Receiver Operating Characteristic (ROC) curves.

## 3. Results

### 3.1. Patients’ Demographic and Clinical Data

A total of 83 patients were included in our study. All 83 patients included in the study underwent (neoadjuvant) chemotherapy after their initial diagnosis. Seventy-three of these patients subsequently underwent surgery. The latter group included 19 (26%) women and 54 (74%) men. The age range at the time of initial diagnosis was 31 to 83 years, with a median of 64 years. Men (Mdn = 65.50) were significantly older than women (Mdn = 59.00) according to the Mann–Whitney U test, U = 699.50, z = 2.35, *p* = 0.019, r = 0.27. Twenty-seven (37%) of the patients had esophageal carcinoma, while 46 (63%) were diagnosed with gastric carcinoma. Tumor staging via TNM classification revealed grade T3 tumor extension in most cases (71.2%), grade N1 lymph node involvement (75.3%), and no distant metastases were present in 93.2% of the cases—grade M0. Due to the presence of liver metastasis, one patient was formally operated at stage M1, and this was resected simultaneously. Patients’ baseline demographic and clinical data are given in Table 3.

### 3.2. Sarcopenia

In total, 249 CT datasets with 996 muscle segmentations were analyzed. As described above, the threshold value of the lowest quartile of the TPI values was determined for each sex at t1, t2 and t3 to define sarcopenia. The calculated values for the patient group “with surgery” are shown in Table 4. Patients whose TPI value was below this threshold were considered sarcopenic. The respective percentages of patients with sarcopenia are given in brackets.

**Figure 3:**
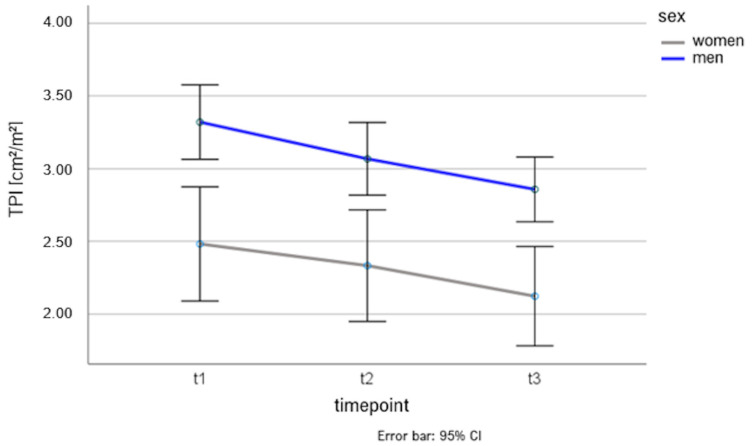
Gender-specific change in TPI as a function of time points. TPI: Total Psoas Index.

Repeat-measurement ANOVA revealed that for the “surgery” group, regardless of gender, TPI decreased significantly from t1 to t2 and t2 to t3 (*p* < 0.001). Men had significantly higher TPI than women at each time point (*p* < 0.001). The rate of decline of TPI between genders was not significantly different (*p* = 0.690). The results are shown graphically in Figure 3.

Fisher’s exact tests did not show at any of the time points—t1 (*p* = 0.465, OR = 0.71) t2 (*p* = 0.342, OR = 0.51) and t3 (*p* = 0.277, OR = 1.82)—that patients with sarcopenia had significantly more PD after tumor therapy than patients without sarcopenia. On average, PD occurred after 271.69 ± 104.20 days. The Mann–Whitney U test showed that the time from initial diagnosis to PD was not significantly shorter in the “sarcopenia” group than in the “non sarcopenia” group at any of the time points. (t1: Mdnsarcopen = 255, Mdnnon_sarcopen = 217, U = 25.00, z = 0.74, *p* = 0.521, r = 0.19; t2: Mdnsarcopen = 224, Mdnnon_sarcopen = 237, U = 12.00, z = −0.32, *p* = 0.817, r = −0.08; t3 = Mdnsarcopen = 255, Mdnnon_sarcopen = 217, U = 33.0, z = 0.62, *p* = 0.583, r = 0.16).

### 3.3. Radiomics

The means and standard deviations of the Accuracy and AUC for the three different algorithms trained in the machine learning models (DT, KNN, RF) were determined. The results related to the radiomic features of one of the three segmented muscles (M. psoas major, M. quadratus lumborum, M. erector spinae) and a defined target parameter were calculated separately. The defined target parameters included sarcopenia, PD and PD in the presence of sarcopenia, so-called sarcopenia + PD.

For the target parameter sarcopenia, the best values at time t1 were shown for the RF for the M musculus psoas major (Accuracy 0.90 ± 0.03; AUC 0.96 ± 0.02) and the Musculus quadratus lumborum (Accuracy 0.89 ± 0.11; AUC 0.95 ± 0.09) followed by the Musculus erector spinae. For the target parameter PD, the best results were shown with the RF model for the quadratus lumborum muscle (Accuracy 0.88 ± 0.04; AUC 0.93 ± 0.04), followed by the erector spinae muscle (Accuracy 0.88 ± 0.06; AUC 0.91 ± 0.04) and the psoas major muscle (Accuracy 0.78 ± 0.09; AUC 0.85 ± 0.09). For the sarcopenia + PD target parameter, the best values were obtained with the RF model for the psoas major muscle (Accuracy 0.93 ± 0.04; AUC 0.97 ± 0.04) and the erector spinae muscle (Accuracy 0.93 ± 0.09; AUC 0.90 ± 0.00), followed by the results for the quadratus lumborum muscle (Accuracy 0.68 ± 0.08; AUC 0.73 ± 0.06).

For the time point t2 and the target parameter sarcopenia, the best values were also evaluated for the psoas major muscle with the RF and DT model (Accuracy 0.86 ± 0.06; AUC 0.91± 0.06). For the target parameter PD, the best results were obtained with the RF model for the quadratus lumborum muscle (0.80 ± 0.05/0.84 ± 0.06). For the target parameter sarcopenia + PD, the best results were shown with the RF model for the erector spinae muscle (0.93 ± 0.09/0.90 ± 0.00).

Results for all machine learning models at time points t1 and t2 for each muscle with the best results are given in Table 5.

**Figure 4:**
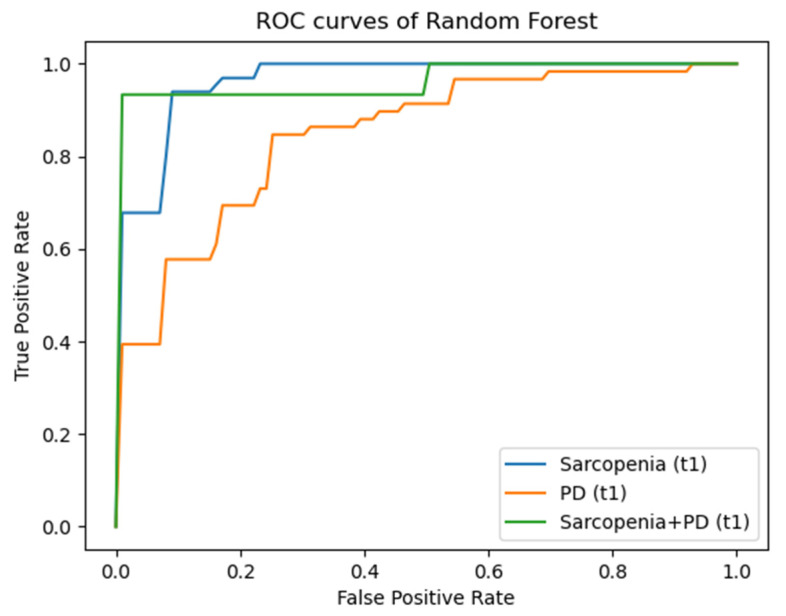
ROC-curves for AUC results for time point t1. ROC: Receiver Operating Characteristic; PD: Progressive Disease.

**Figure 5:**
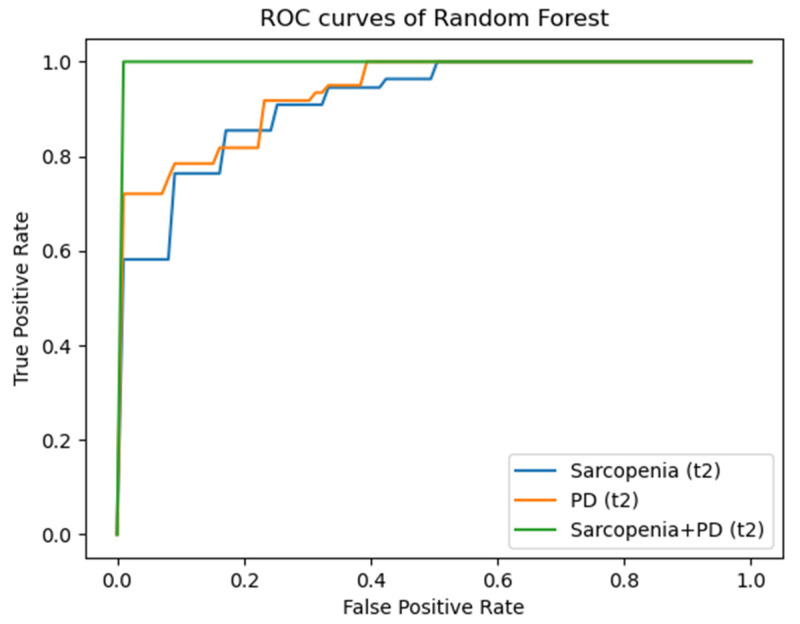
ROC-curves for AUC results for time point t2. ROC: Receiver Operating Characteristic; PD: Progressive Disease.

**Figure 6:**
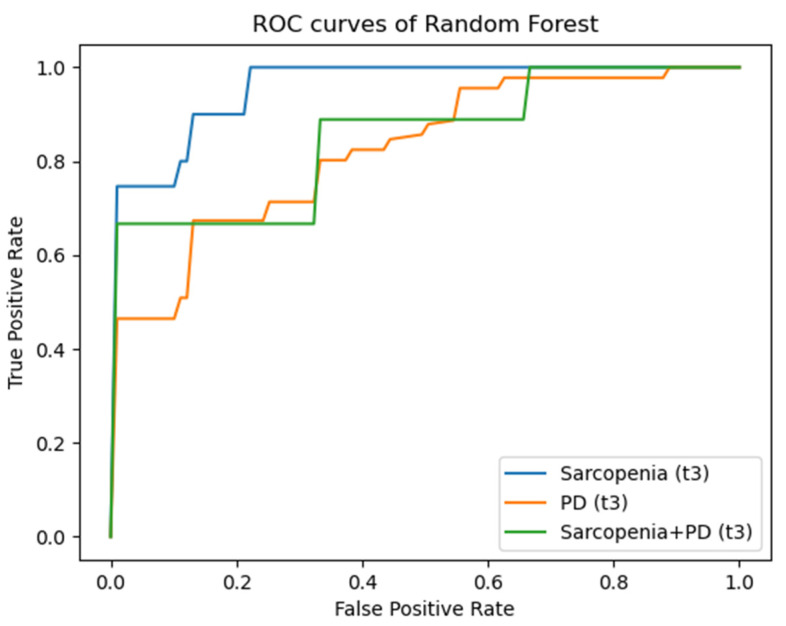
ROC-curves for AUC results for time point t3. ROC: Receiver Operating Characteristic; PD: Progressive Disease.

Figure 4, Figure 5 and Figure 6 illustrate the AUC results of the RF models based on the radiomic features of the psoas major muscle for the entire patient collective for the time points t1 to t3 using ROC curves as examples. Here, each of the figures comprises three graphs, each representing the mentioned outcomes with respect to one of the defined target parameters (sarcopenia, PD, sarcopenia + PD). An imaginary diagonal between the x- and the y-axis, which represents the same ratio of true positive and false positive predictions, i.e., a purely random basis, clarifies that all shown curves lie above this diagonal. The clear distance of the curves from this imaginary diagonal in the direction of the left and upper boundary line of the enclosing square, which can be recognized without exception, visually illustrates the prognostic and diagnostic significance of the developed machine learning models, which have already been described in the previous sections. At time t3, one can comparatively see the smallest distance of the curves from the random diagonal, especially with regard to the target parameter PD—both in the entire patient collective and in the collective of patients exhibiting sarcopenia.

## 4. Discussion

Sarcopenia has been considered a prognostic risk factor for tumor patients for several years. The fact that tumors of the stomach and esophagus lead to a particularly unfavorable risk situation, increased postoperative complication rate and reduced overall survival due to the combination of reduced muscle mass and strength as a result of physical inactivity during therapy and additionally restricted nutritional possibilities due to the prominent tumor location, has already been shown and reported in recent studies [13,51]. In clinical practice, it is important to be able to define the risk of tumor patients as efficiently and early as possible, while not ignoring the influence of concomitant diseases. In our study, CT data sets from the regular tumor staging were investigated for their potential to provide well-founded bases and indications with regard to the diagnosis of sarcopenia and the one-year follow-up period. For this purpose, conventional methods of radiological imaging as well as previously established machine learning methods based on radiomic features were applied and tested for their informative value.

### 4.1. Assessment of Sarcopenia

In the present study, the TPI was chosen to define sarcopenia. The values for the TPI evaluated in our study appear to be lower than those otherwise reported in the literature [31,32]. This is possibly due to the small study population. For this reason, we decided to use the threshold value at the lowest gender-specific quartile at the respective time points (t1 to t3) as the threshold value for the diagnosis of “sarcopenia”. The method proved to be clinically feasible. This diagnostic method was also chosen and tested in other studies [35,36,37,39,40]. The validity of the TPI as the sole diagnostic index for sarcopenia and the use of the quartile threshold is critically discussed at times. By using the quartile threshold, about a quarter of male and female patients are described as sarcopenic without any other evidence. For sarcopenia diagnosis, not only the TPI but often the total skeletal muscle area at the L3 level in terms of a skeletal muscle index (SMI) is used. In their often-cited study, Prado et al. investigated the prevalence of sarcopenia in patients with respiratory and gastrointestinal tract tumors and described an overall prevalence of 15%. For this purpose, they generated sex-specific cut-off values to define sarcopenia, which described a significant correlation between low muscle mass and mortality. The values were based on data from patients with sarcopenic obesity [52]. A patient population with the same tumor diseases was analyzed by Martin et al. [8]. They also proposed gender-specific cut-off values based on the studied data, adjusted for the different BMI categories, describing the association between the variables muscle mass and survival, and reported prevalence values of 53% in women and 31% in men based on these values. Although indicated in some studies, there are no generally accepted cut-off values for the diagnosis of sarcopenia.

The variability of existing methods and cut-off values in the literature illustrates the lack of a uniform diagnostic process for sarcopenia. The classification and analysis of a patient population can only be done after consideration of the most appropriate or feasible method and can only be compared with other studies under consideration of methodological differences.

Establishing uniform cut-off values that account for gender as well as age groups and the influence of different disease patterns is a comprehensive and possibly impossible task. However, it is important to further define and test diagnostic cut-off points for specific patient groups.

### 4.2. Effect of Sarcopenia

Statistical analysis to determine the effect of sarcopenia on patient outcomes one year after surgery did not yield a significant result in the present study. Thus, patients who were considered sarcopenic did not experience PD more often or earlier than patients who were not diagnosed with sarcopenia. Based on their analysis of a patient population of 279 patients with gastric cancer, Shi et al. reported a nearly 50% higher risk of postoperative complications and a prolonged hospital stay—albeit by a few days—after gastrectomy for patients with sarcopenia compared to patients without sarcopenia, according to their definition based on both TPI and SMI [53]. The authors did not take a look at the duration of recurrence-free survival as in the present study. In their analyses, Martin et al. also found prognostically unfavorable correlations in their patient collective of over 1500 patients with tumors in the lung or gastrointestinal tract: they reported shorter survival times for the tumor patients with sarcopenia, defined according to their threshold values [8]. However, Martin et al. did not consider sarcopenia alone as a prognostic factor, as in the present study, but in their multivariate analysis, they simultaneously considered the influence of the factors BMI, weight loss, and muscle density, also measured on the CT dataset, which seems to be a promising approach. O’Brien et al. analyzed an adverse significant association between sarcopenia and the incidence of postoperative complications and overall survival in their patient population of 56 patients with gastric cancer [12]. As in the present study, the authors reported that there was no significant difference with regard to recurrence-free survival in the groups with and without sarcopenia. Elliott et al. followed their patient population of 261 patients with esophageal cancer over the course of neoadjuvant therapy, similar to the present study [54]. They reported partly comparable and partly contrasting results. They also showed no significant association between sarcopenia diagnosed at baseline and disease progression after neoadjuvant therapy, which can be compared with the fact that the present study also showed no association between sarcopenia at t1 and PD. However, sarcopenia was associated with disease progression after neoadjuvant therapy in their study. Similarly, they found a significant association between sarcopenia and postoperative complications and reduced disease-specific survival.

For example, the generally recurring tendency for sarcopenia to show a detrimental effect with respect to surgical success and postoperative recovery as well as survival can be attributed, in addition to the underlying disease, to overall additional reduced physical resources due to decreased muscle quantity and quality. Both the TPI and the SMI have already proven to be suitable prognostic parameters. In order to further filter out the best variant in the future, the TPI and the SMI should not only be compared as diagnostic parameters but should also be tested for their correlation with regard to prognostic target parameters, or directly compared.

### 4.3. Radiomics

The machine learning models developed in the present study were tested for their performance using the metrics Accuracy and AUC in order to be able to make statements about the diagnostic and prognostic value of the radiomic features with regard to sarcopenia and tumor progression. The analyses were performed not only on the total population but also on subgroups—as in the statistical analysis in the course of conventional sarcopenia diagnostics—in order to detect differences due to demographic and clinical influencing factors. The performance of the machine learning models can be evaluated as promising in an overview of all obtained results: It is shown that the models developed here were able to diagnose or predict both sarcopenia and PD based on the radiomic features of examined muscles.

A closer look at the algorithms investigated in the different models showed that, regardless of the time point t1 to t3 defined in this study, the muscle considered, and the target parameter defined, values between 0.7 and 1.0 were calculated for the AUC of the RF model. Thus, the highest values were found here compared with the other models. In addition, consistently good and satisfactory values were seen based on the radiomic features of the psoas major muscle, although they shifted away from the AUC of the RF toward the results of the DT.

To date, only a few studies have used radiomic features in the context of sarcopenia. In their study, Dong et al. investigated the possibility of identifying sarcopenia in patients with lung cancer based on radiomic features of a pretherapeutic axial chest CT using a machine learning model [26]. Unlike the present study, the authors chose the complete SMA of the CT cross-section as the ROI, in addition to a deviated CT plane, and used the cut-off values for the diagnosis of sarcopenia proposed by Martin et al. [8]. Before training their model, the authors selected the generated radiomic features, excluding redundant features. Their model was based on a novel extension of a DT. To evaluate the performance of their developed model, the authors likewise selected Accuracy and AUC, among others. On this basis, Dong et al. achieved values of 0.93 for both Accuracy and AUC. In comparison with the results of the present study, which ranged from 0.70 to 0.85 in the DT model with the target parameter sarcopenia regardless of the time point considered, the values of Dong et al. perform better at this point. It should be noted, however, that the results are difficult to compare—for example, due to the different tumor entities of the two patient collectives as well as the different processing of the CT data sets and different implementation of the machine learning method. Presumably, however, the feature selection made by Dong et al. also has a decisive influence on the results. Chen et al. investigated the suitability of radiomic features for the pre-operative diagnosis of sarcopenia in patients with gastric cancer and its predictive value with regard to postoperative complications and patient survival [55]. To define sarcopenia, the authors used the SMA at the level of the lumbar spine and additionally included the factors of muscle strength or physical capacity. Radiomic features were collected from a three-dimensional measurement but were limited to the psoas major muscle. Statistical analysis procedures were used to select features that showed association with sarcopenia defined by conventional means and then incorporated into the model. The performance or accuracy of the model was checked using the AUC and yielded a value of 0.83 for the test group for the diagnosis of sarcopenia, which is comparable to the results of the present study based on the psoas major muscle with the target parameter of sarcopenia, especially with the values of the DT model, regardless of the model selected.

In summary, the various approaches to integrating radiomic features into diagnostic and prognostic processes in everyday clinical practice have already yielded some promising results to date. However, Chen et al., as well as Sah et al., have already emphasized in their reviews, which do not address sarcopenia but the potential of radiomic features in the context of tumor diseases, the importance of the necessity of standardization in order to reliably integrate the radiomic features into clinical processes, respectively [56,57]. With more studies according to a standardized and thus comparable protocol, a specific selection of features could possibly emerge that represents a suitable data basis for sarcopenia or a specific tumor entity with regard to diagnostics and prognostics, which can then be focused on during feature extraction. The choice of machine learning model and the workflows for developing these models should also be standardized.

### 4.4. Limitations

The retrospective approach of the present study is certainly a limiting factor. To enhance the validity and significance of explored influences and dependencies, ensuring homogeneity in the patient population is crucial in future prospective studies. This entails achieving a balanced gender distribution and comparable underlying tumor stages among patients. The heterogeneous nature of tumor stages in the current study leaves open the possibility that the initial tumor stage might indeed impact both sarcopenia and the disease progression. The definition of sarcopenia used in this study must also be considered limiting: Sarcopenia was defined using the lowest gender-specific quartile threshold at the respective time points. This threshold is specific to our analyzed population. Furthermore, the TPI values observed in our study were lower compared to other study populations. Future studies should consider analyzing both TPI and SMI simultaneously to better dichotomize sarcopenia, enabling the identification of the most appropriate method. In addition, based solely on the measurement of CT data sets, the change in muscle function is not considered an additional diagnostic criterion. However, since the diagnosis of tumor patients does not include the examination of muscle function, this approach is the only way to retrospectively analyze sarcopenia, and it allows for the comparison of different studies that have often defined sarcopenia in this way. For future studies, a prospective study approach that includes the examination of muscle function is recommended in order to consider the currently valid complete definition when diagnosing sarcopenia.

## 5. Conclusions

In conclusion, it can be stated that with regard to the diagnosis of sarcopenia and the prognosis of tumor progression during the one-year postoperative course in patients with gastric and esophageal carcinomas, the CT radiomics of skeletal muscle together with machine learning is correlated with the presence of sarcopenia and can additionally assist in predicting disease progression. In the present study, although no significant association between sarcopenia diagnosed with PMI and tumor progression was found, both the method based on muscle segmentation in the context of conventional radiological diagnostics and the possibilities of machine learning based on radiomics offer the potential for application in clinical practice.

In conclusion, it can be stated that with regard to the diagnosis of sarcopenia and the prognosis of tumor progression during the one-year postoperative course in patients with gastric and esophageal carcinomas that CT radiomics of skeletal muscle together with machine learning, correlated with the presence of sarcopenia and can additionally assist in predicting disease progression. In the present study, although no significant association between sarcopenia diagnosed with PMI and tumor progression was found, both the method based on muscle segmentation in the context of conventional radiological diagnostics and the possibilities of machine learning based on radiomics offer the potential for application in clinical practice.

## Figures and Tables

**Table 1:** Settings of the radiomics feature extraction.

Setting	Determination
Bin Method	FBN
Bin Amount	32
LoG Filter	0
LoG Sigma	2
Matrix Aggregation	3D Average
Method	Directions
Resample Filter	1
Resample Spacing X	1
Resample Spacing Y	1
Resample Spacing Z	1
Second-Order Distance	1
Threshold Filter	0

**Table 2:** Radiomics features selected by LASSO.

Radiomics Features of First Order	Radiomics Features of Second Order: Gray Level Co-Occurrence Matrix (GLCM)
Area	Clustershade (GCS)
Maximum Histogram Gradient (HMXG)	
Histogram P10th (HP10)	
Histogram Robust Mean Absolute Deviation (HRMAD)	
Intensity Interquartile Range (IIQR)	
Intensity Mean (IMN)	
Intensity P10th	

**Table 3:** Patients’ demographic and clinical characteristics at the time of diagnosis. *n*: total number of patients; T: primary tumor; N: lymph nodes; M: metastasis.

Characteristics	Patients with Surgery (*n* = 73)	Patients without Surgery (*n* = 10)
Sex		
Female	19 (26%)	5 (50%)
Male	54 (74%)	5 (50%)
Age total	64 (31–83)	65 (40–75)
Age female	59 (31–77)	63 (40–75)
Age male	65.5 (36–83)	67 (49–75)
≤65	42 (57.5%)	5 (50%)
<65	31 (42.5%)	5 (50%)
Tumor		
Gastric cancer	46 (63%)	4 (40%)
Esophageal cancer	27 (37%)	6 (60%)
T stage		
T0	1 (1.4%)	0 (0%)
T1	1 (1.4%)	1 (10%)
T2	11 (15.1%)	2 (20%)
T3	52 (71.2%)	7 (70%)
T4	8 (11%)	0 (50%)
N stage		
N0	9 (12.3%)	2 (20%)
N1	55 (75.3%)	7 (70%)
N2	6 (8.2%)	1 (1%)
N3	3 (4.1%)	0 (0%)
M stage		
M0	72 (98.6%)	6 (60%)
M1	1 (1.4%)	4 (40%)

**Table 4:** Gender specific threshold values for sarcopenia diagnosis for the different time points of analysis. The numbers in parentheses indicate the percentage of patients with sarcopenia.

Time Point	Female	Male
t1	1.92 cm^2^/m^2^ (21.1%)	2.59 cm^2^/m^2^ (24.1%)
t2	1.89 cm^2^/m^2^ (21.1%)	2.16 cm^2^/m^2^ (22.2%)
t3	1.74 cm^2^/m^2^ (15.8%)	2.28 cm^2^/m^2^ (20.4%)

**Table 5:** Results for the machine learning models for the radiomic features for time points t1 and t2. For each time point and target parameter, the best result is illustrated. M: musculus; PD: progressive disease; DT: decision tree; KNN: K-nearest to neighbor; RF: random forest; AUC: area under the curve.

Time Point	Muscle	Target Parameter	DT	KNN	RF
			Accuracy/AUC	Accuracy/AUC	Accuracy/AUC
T1	M. psoas	Sarcopenia	0.89 ± 0.05/0.89 ± 0.04	0.87 ± 0.08/0.93 ± 0.04	0.90 ± 0.03/0.96 ± 0.02
T1	M. qu. lumb.	PD	0.84 ± 0.05/0.86 ± 0.07	0.84 ± 0.03/0.85 ± 0.03	0.88 ± 0.04/0.93 ± 0.04
T1	M. psoas	Sarcopenia + PD	0.90 ± 0.00/0.90 ± 0.00	0.84 ± 0.15/0.85 ± 0.13	0.93 ± 0.04/0.97 ± 0.04
T2	M. psoas	Sarcopenia	0.86 ± 0.10/0.91 ± 0.08	0.77 ± 0.10/0.77 ± 0.10	0.86 ± 0.10/0.91 ± 0.08
T2	M. qu. lumb.	PD	0.74 ± 0.07/0.74 ± 0.08	0.73 ± 0.09/0.73 ± 0.11	0.80 ± 0.05/0.84 ± 0.06
T2	*M. erector* sp.	Sarcopenia + PD	0.86 ± 0.04/0.87 ± 0.03	0.82 ± 0.19/0.89 ± 0.12	0.93 ± 0.09/0.90 ± 0.00

## Data Availability

Data is contained within the article.

## Appendix A

**Table A1:** Radiomics features extracted for model development.

Radiomics Features of First Order	Radiomics Features of Second Order: Gray Level Co-Occurrence Matrix (GLCM)
Histogram Minimum	Joint Maximum
Histogram Maximum	Joint Average
Histogram Range	Standard Deviation
Histogram Mean	Joint Variance
Histogram Variance	Joint Entropy
Histogram Standard Deviation	Difference Average
Histogram Skewness	Difference Variance
Histogram Kurtosis	Difference Entropy
Histogram Entropy	Sum of Averages
Histogram Uniformity	Sum of Variance
Histogram Mean Absolute Deviation	Sum of Entropy
Histogram Robust Mean Absolute Deviation	Angular Second Moment
Histogram Median Absolute Deviation	Contrast
Histogram Coefficient Variation	Dissimilarity
Histogram Quartile Coefficient Dispersion	Inverse Difference
Histogram Interquartile Range	Inverse Difference Normalized
Histogram P10th	Inverse Difference Moment
Histogram P25th	Inverse Difference Moment Normalized
Histogram P50th	Joint Maximum
Histogram P75th	Joint Average
Histogram P90th	Standard Deviation
Histogram Minimum Histogram Gradient Intensity	Joint Variance
Histogram Maximum Histogram Gradient Intensity	Joint Entropy
Intensity Minimum	Difference Average
Intensity Maximum	Difference Variance
Intensity Range	Difference Entropy
Intensity Mean	Sum of Averages
Intensity Variance	Sum of Variance
Intensity Standard Deviation	Sum of Entropy
Intensity Skewness	Angular Second Moment
Intensity Kurtosis	Contrast
Intensity Energy	Dissimilarity
Intensity P10th	Inverse Variance
Intensity P25th	Correlation
Intensity P50th	Auto Correlation
Intensity P75th	Cluster Shade
Intensity P90th	Cluster Prominence
Intensity Root Mean Square	Cluster Tendency
Intensity Mean Absolute Deviation	Information Correlation 1
Intensity Robust Mean Absolute Deviation	Information Correlation 2
Intensity Median Absolute Deviation	Inverse Variance 41
Intensity Coefficient Variation	
Intensity Quartile Coefficient Dispersion	
Intensity Interquartile Range 44	
